# Traumatic Brain Injury during the SARS-CoV-2 Pandemic

**DOI:** 10.1089/neur.2020.0001

**Published:** 2020-07-23

**Authors:** Juliette A.L. Santing, Crispijn L. van den Brand, Korné Jellema

**Affiliations:** ^1^ Department of Neurology, Haaglanden Medical Center, The Hague, The Netherlands.; ^2^ Department of Emergency Medicine, Haaglanden Medical Center, The Hague, The Netherlands.; ^3^ Dutch Institute for Clinical Auditing, Leiden, The Netherlands.

**Keywords:** coronavirus, COVID-19, head trauma, SARS-CoV-2, traumatic brain injury

## Abstract

Emergency departments (EDs) are eerily quiet for illnesses apart from COVID-19. In this short communication, we assessed the effect of COVID-19 on ED attendance rates for traumatic brain injury (TBI). Data were collected from all consecutive patients with TBI attending our hospital (Haaglanden Medical Center, The Hague, The Netherlands) during the first 3 weeks of the Dutch lockdown (from March 18 to April 6) and for the same period last year. We observed a 36% decrease in ED attendance for TBI since the beginning of the SARS-CoV-2 pandemic (91 vs. 143). Patients who presented during the lockdown were significantly older compared with the patients who visited the ED in the previous year (72 vs. 57, *p* = 0.01). No other significant differences were found.

## Introduction

COVID-19 has a profound effect on emergency department (ED) visits. Clinicians worldwide are facing a far lower demand than normal for emergency treatment, apart from COVID-19. The first official Public Health England data since the lockdown began revealed a 29% year-on-year drop in ED attendance, whereas emergency admissions showed a sharp fall, down by 23%.^1^

Traumatic brain injury (TBI) is one of the most common reasons for ED department visits around the world.^2.^It is responsible for 2.5 million ED visits in the United States and 1.4 million in the United Kingdom annually.^2,3^ Around 80% of patients with TBI have mild TBI, classified by a Glasgow Coma Scale (GCS) score ranging from 13 to 15.^4^ Overall, only a minority of cases, ranging from 5 to 10%, harbor an acute intracranial injury, which for the large majority are represented by intracranial hemorrhages.^5^

Hospital across The Netherlands are reporting fewer heart attacks, acute strokes, and other medical emergencies. We hypothesized that the “coronavirus lockdown effect” also is present on ED attendance rates for TBI. However, clinical registry data confirming this trend and exploring possible reasons have been lacking. Therefore, the aim of this short communication was to assess the change in the number of patients with TBI presenting to the ED during the SARS-CoV-2 pandemic and to identify potential clinical predictors for the probability of ED attendance related to TBI.

## Methods

We collected data from consecutive patients of all ages with TBI presenting to our Level 1 trauma center during the first 3 weeks of the Dutch lockdown (from March 18 to April 6) and for the same period last year. Demographic characteristics, risk factors for intracranial complications, and traumatic intracranial findings were collected. Risk factors for intracranial complications included use of antithrombotic agents; loss of consciousness (LOC) reported by the patient or witness; post-traumatic amnesia (PTA) reported by the patient, witness, or tested at neurological examination; trauma mechanism; vomiting; signs of a skull base fracture; visible injury to the head (excluding the face); and neurological deficit.

### Statistical analysis

IBM SPSS Statistics (IBM, Armonk, NY, USA) version 24 for Windows was used to perform the statistical analysis. Binary data were described as numbers and percentages, and continuous data were presented as median values with interquartile ranges (IQRs). To evaluate differences between groups the chi-square test was used for categorical data and the Mann-Whitney U test for continuous data. A *p*-value <0.05 was considered as statistically significant.

## Results

The total number of attendances during the first 3 weeks of the lockdown was 91, which was 36% lower than in the same period the year before. Patients who presented to the ED during the lockdown were significantly older compared with the patients who visited the ED in the previous year (72 vs. 57, *p* = 0.01). We observed a net reduction in all the mechanisms of injury. There was a trend toward more use of antithrombotic agents among the group presenting during the lockdown (*p* = 0.05). Sex, intracranial bleeding prevalence, admission GCS scores, and other risk factors of intracranial complications were not significantly different between the groups (Table 1).

**Table 1.** Patient Characteristics

Variable	2019 (n = 143)	2020 (n = 91)	P-value
Age, years			
Median (IQR)	57 (32–76)	72 (37–83)	0.01^a^
Range	0–99	0–98	
<25, *n* (%)	25(17.5)	13 (14.3)	0.52^b^
25–50, *n* (%)	35 (24.5)	15 (16.5)	0.15^b^
50–75, *n* (%)	44 (30.8)	24 (26.7)	0.47^b^
>75, *n* (%)	39 (27.3)	39 (42.9)	0.01^b^
Men, *n* (%)	75 (52.4)	53 (58.2)	0.38^b^
Use of antithrombotic agents, *n* (%)	43 (30.1)	39 (42.9)	0.05^b^
Intracranial hemorrhage, *n* (%)	4 (2.8)	3 (3.3)	0.83^b^
Mechanism of injury, *n* (%)^c^			
Falls	88 (61.5)	63 (69.2)	0.23^b^
Bicycle related	11 (7.7)	7 (7.7)	0.9^b^
Motor vehicle traffic	16 (11.2)	6 (6.6)	0.24^b^
Struck by/against	13 (9.1)	4 (4.4)	0.18^b^
Assault	13 (9.1)	8 (8.8)	0.94^b^
Alcohol intoxication, *n* (%)	10 (7.0)	8 (8.8)	0.62^b^
Classification of head injury, *n* (%)			
Mild	138 (96.5)	90 (98.9)	0.26^b^
Moderate	1 (0.7)	1 (0.7)	0.75^b^
Severe	4 (2.8)	0 (0.0)	0.11^b^
LOC, *n* (%)^d^	21 (14.7)	8 (8.8)	0.17^b^
PTA, *n* (%)^e^	16 (11.2)	8 (8.8)	0.52^b^
Anisocoria, *n* (%)	2 (1.4)	0 (100)	0.26^b^
Vomiting, *n* (%)	5 (3.5)	8 (8.8)	0.09^b^
Neurological deficit, *n* (%)	2 (1.4)	1 (1.1)	0.84^b^
Visible injury of the head, *n* (%)	99 (69.2)	71 (78.0)	0.14^b^
Signs of skull fracture, *n* (%)	2 (1.4)	1 (1.1)	0.84^b^

^a^ Determined by use of the Mann-Whitney U test; ^b^determined by use of the chi-squared test; ^c^missing *n* = 5, 2.1%; ^d^missing *n* = 14, 6%; ^e^missing *n* = 7, 3%.

IQR, interquartile range; LOC, loss of consciousness; PTA, post-traumatic amnesia.

## Discussion

We observed at our hospital in The Netherlands a 36% decrease in ED attendances for TBI during the lockdown compared with the same period last year. This finding is in concordance with the fall in the number of individuals seeking help for other urgent conditions. The reason for the sharp drop-off might be explained by a combination of factors. First, there are less accidents due to the stay-at-home orders. Second, some patients express reluctance to visit a hospital due to fear of being exposed to the virus. Third, some patients did not want to pose an extra burden on the health service. Last, other factors might include a change in outpatient referrals and stricter triage in the ED.

The fall in total ED attendances appears to be most marked in people from 5 to 44 years of age.^1^ We observed a trend toward a decline in ED attendance in people from 25 to 50 years of age. The reduction in the number of accidents in these age groups is probably a result of less commuting; closing of restaurants, bars, and schools; fewer social events; and reduced sport-related activities.

This investigation has limitations and its results should be interpreted in light of them. First, the study was designed for rapid patient accrual and data collection in an urgent global crisis. Therefore, the time frame of our study is relatively short and the overall sample size is somewhat small. A multi-center approach or longer inclusion period would probably have resulted in more robust findings. Nevertheless, we found a significant decline in ED attendance for TBI during the lockdown compared with the same period last year. Second, the retrospective nature of the study exposes it to the bias appropriate to its design and some information may be missing. However, the clinical protocol used in the ED requires the clinician to carefully evaluate the pre- and post-traumatic risk factors presented by the patient, and therefore we believe that the information is accurate. Finally, although we observed a possible reluctance of patients to present to hospitals, we were unable to confirm this with individual patients. It needs to be mentioned that we describe ED attendance rates for TBI in the early phases of the pandemic when attendance was not influenced by the appeal of medical professionals to continue using emergency facilities and the loosening of behavioral measures such as social distancing, fewer visits with family, avoiding visits to elderly/fragile people, and following hygiene measures that occurred when the lockdown continued.

## Conclusion

Our results show a 36% decrease in ED attendances for TBI, in line with the fall in numbers of patients presenting with other serious medical conditions since the beginning of the SARS-CoV-2 pandemic.

## Abbreviations Used

ED: emergency department
GCS: Glasgow Coma Scale
IQR: interquartile range
LOC: loss of conciousness
PTA: post-traumatic amnesia
TBI: traumatic brain injury
